# Ex vivo ultrasonic samples of human brain tumors in the molecular era

**DOI:** 10.1093/noajnl/vdaa014

**Published:** 2020-02-08

**Authors:** Alastair J Kirby, José P Lavrador, Istvan Bodi, Francesco Vergani, Ranjeev Bhangoo, Keyoumars Ashkan, Gerald T Finnerty

**Affiliations:** 1 Department of Basic and Clinical Neuroscience, King’s College London, London, UK; 2 Department of Neurosurgery, King’s College Hospital NHS Foundation Trust, London, UK; 3 Department of Clinical Neuropathology, King’s College Hospital NHS Foundation Trust, London, UK; 4 Department of Neurology, King’s College Hospital NHS Foundation Trust, London, UK

**Keywords:** glioma, molecular marker, rotoporphyrin IX, tumor heterogeneity, 5-aminolevulinic acid

## Abstract

**Background:**

Gliomas are composed of multiple clones of tumor cells. This intratumor heterogeneity contributes to the ability of gliomas to resist treatment. It is vital that gliomas are fully characterized at a molecular level when a diagnosis is made to maximize treatment effectiveness.

**Methods:**

We collected ultrasonic tissue fragments during glioma surgery. Large tissue fragments were separated in the operating theater and bathed continuously in oxygenated artificial cerebrospinal fluid to keep them alive. The ex vivo tissue fragments were transferred to a laboratory and incubated in 5-aminolevulinic acid (5-ALA). 5-ALA is metabolized to Protoporphyrin IX (PpIX), which accumulates in glioma cells and makes them fluorescent. The molecular and neuropathological features of the PpIX fluorescent ultrasonic tissue fragments were studied.

**Results:**

We show that PpIX fluorescence can rapidly identify tissue fragments infiltrated by glioma in the laboratory. Ultrasonic tissue fragments from the tumor core provided molecular and neuropathological information about the glioma that was comparable to the surgical biopsy. We characterized the heterogeneity within individual gliomas by studying ultrasonic tissue fragments from different parts of the tumor. We found that gliomas exhibit a power relationship between cellular proliferation and tumor infiltration. Tissue fragments that deviate from this relationship may contain foci of more malignant glioma. The methylation status of the *O*^6^-methylguanine DNA methyltransferase gene promoter varied within each glioma.

**Conclusions:**

Ex vivo ultrasonic tissue fragments can be rapidly screened for glioma infiltration. They offer a viable platform to characterize heterogeneity within individual gliomas, thereby enhancing their diagnosis and treatment.

**Key Points**

- 5-ALA-induced fluorescence detects glioma infiltration in ex vivo brain tissue.
- Ultrasonic tissue fragments from gliomas provide diagnostic molecular information.
- 5-ALA-screened ultrasonic tissue fragments can reveal intratumoral heterogeneity.

**Importance of the Study**

Gliomas are comprised of multiple clones of tumor cells. This intratumor heterogeneity is a key factor in the ability of gliomas to resist treatment. It is vital that gliomas have a full molecular characterization at diagnosis to maximize treatment efficacy. We developed a rapid fluorescence-based screening protocol to identify glioma infiltration in ex vivo brain tissue fragments collected by ultrasonic aspirators. We show that brain tissue fragments provide molecular information that is relevant to diagnosis and treatment. We propose that ex vivo ultrasonic tissue fragments are a valuable resource that can be used to assess intratumoral heterogeneity.

Gliomas were originally classified according to their histopathological features. Recently, however, greater emphasis has been placed on the molecular profile of gliomas for diagnosis and prognosis.^1^ In parallel, precision medicine treatments of gliomas are being based on the molecular profiles of the glioma cells.

Research studies have employed molecular profiling to show that gliomas do not have a uniform cellular composition, but rather are composed of multiple cell types at different stages of differentiation.^2–5^ Therefore, a neuropathological diagnosis of gliomas is most accurate when multiple sites within the tumor are sampled.^6^ This presents a problem when the tumor is near an eloquent area or is difficult to access surgically.

Ultrasonic aspirators generate small fragments of tissue that are aspirated as they are produced.^7^ These devices can remove tumor tissue while leaving neighboring, healthy brain parenchyma and blood vessels intact. This feature combined with their ease of use meant that they were rapidly adopted by neurosurgeons for microsurgical dissection of brain tumors.^8^

It was quickly realized that the tissue fragments generated by ultrasonic aspirators may have diagnostic value. Concerns were raised that the small samples produced by ultrasonic aspirators may not show key histopathological features, such as necrosis, and that the process of fragmenting the tissue may cause artefacts.^9^ However, other studies of brain tumor samples produced by ultrasonic aspirators indicate that they recapitulate many of the histopathological^10^ and immunocytochemical^11^ features found in the larger samples collected with brain tumor biopsy forceps. Moreover, ultrasonic aspirators make it very easy to take samples from multiple parts of the tumor. Despite this, ultrasonic sampling of brain tumors is not in widespread, clinical use.

The ability to screen ultrasonic tissue fragments for tumor infiltration would greatly increase their clinical value. Fluorescence imaging has been used to detect tumor per-operatively.^12–16^ We used the same approach to identify ultrasonic tissue fragments infiltrated with glioma. These ultrasonic tissue fragments were stored under acute conditions used by neuroscientists to keep brain tissue alive.^17,18^ We found that the ultrasonic samples recapitulated the molecular features of the biopsy samples that are collected routinely. Furthermore, ultrasonic samples taken from different parts of the glioma showed heterogeneity in the methylation status of the *O*^6^-methylguanine DNA methyltransferase (*MGMT*) gene promoter and cellular proliferation indices. Our findings suggest that ultrasonic samples can play a valuable role as an adjunct to brain tumor biopsy in both diagnosis and planning treatment.

## Materials and Methods

### Ethics Approval and Consent to Participate

The UK Human Research Authority (https://www.hra.nhs.uk/) approved the collection of brain samples following a favorable opinion from the South West Research Ethics Committee (REC approval code: 18/SW/0022). Brain samples were donated by participants with a suspected diffuse astrocytic or oligodendroglial tumor^1^ who had not had brain surgery previously or received chemotherapy or radiotherapy. All participants gave informed consent prior to their surgery. The consent included permission to use and present their data.

### Intraoperative 5-ALA-Induced Fluorescence

All participants were given 20 mg/kg of 5-ALA 2 h prior to their craniotomy for fluorescence-guided tumor resection at King’s College Hospital, London between January 2019 and September 2019. The neurosurgeon recorded whether (s)he could see 5-ALA-induced fluorescence with the operating microscope (Zeiss OPMI Pentero 900 or Zeiss KINEVO 900 operating microscope) during the surgery.

### Intraoperative Sample Collection

**Fig. 1:**
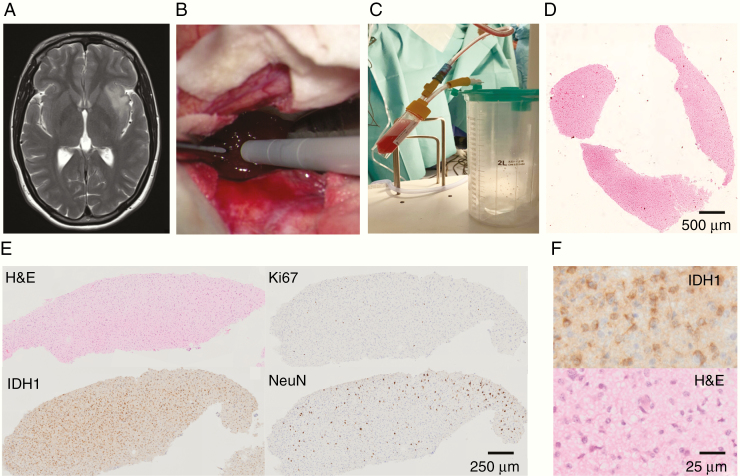
Collection and staining of large ex vivo ultrasonic tissue fragments (UTFs). (A–F) Data from participant A only. (A) T2-weighted MRI of low-grade glioma in left insula. (B) Surgical aspiration of UTFs. (C) Trap for UTF collection in situ. (D) Brightfield image, H&E stained sections of UTFs. (E) Sections from one UTF in panel D stained for H&E, Ki-67, IDH1 (R132H) mutation, and NeuN. (F) Higher magnification images of H&E (top) and IDH1 (R132H) mutation (bottom) sections shown in panel E.

Ultrasonic samples were collected during surgery with a Sonopet ultrasonic aspirator (Stryker Corp.) with an angled handpiece and straight, soft tissue tip (outer diameter 1.92 mm, inner diameter 1.50 mm) oscillating at 25 kHz. Tissue fragments were collected with the following console settings: aspiration 5–10%, power 5–10%, and irrigation 5–10%. The anatomical location of the tissue samples was documented with either the intraoperative surgical navigation system (Stealth S7 or S8, Medtronic) or intraoperative ultrasound (ESAOTE), combined with intraoperative images (Zeiss OPMI Pentero 900 or Zeiss KINEVO 900 operating microscope) of the exposed brain (Figure 1A and B). The tissue fragments were collected in a specimen trap (Pennine, MST-3070) connected to the Sonopet aspiration system (Figure 1C). The tumor biopsy was given preference when the tissue was collected as the tumor biopsy was used for diagnosis and the ultrasonic samples were not.

The operating surgeon co-ordinated the collection of the ultrasonic samples to ensure tumor samples from known locations were sampled. When the specimen trap was filled, the contents were passed through a nylon mesh to separate larger tissue fragments from the smaller fragments. The larger fragments were rinsed with cerebrospinal fluid (CSF) that had been modified to prevent neural firing and excitotoxicity.^19^ The transportation CSF comprised (in mM) 108 choline-Cl, 3 KCl, 26 NaHCO_3_, 1.25 NaHPO_4_, 25 d-glucose, 3 Na pyruvate, 2 CaCl_2_, 1 MgCl_2_, and 1 heparin, which had been cooled to 4°C and bubbled with 95% oxygen/5% CO_2_. The large tissue fragments were then transferred to an ex vivo tissue transportation system, which keeps the tissue fragments cool while bathing them in transportation CSF.^20^

After arriving in the laboratory, the ultrasonic samples were transferred to an incubation chamber. The artificial CSF used for the incubation chamber was changed to (mM): 120 NaCl, 3 KCl, 23 NaHCO_3_, 1.25 NaHPO_4_, 10 d-glucose, 2 CaCl_2_, and 1 MgCl_2_ bubbled with 95% oxygen/5% CO_2_. The artificial CSF was gradually warmed to 37°C. The larger fragments were imaged and then processed for a neuropathological and molecular diagnosis (Figure 1D–F).

### Ex Vivo Imaging

PpIX photobleaches quickly.^21^ This makes it harder to see low levels of PpIX fluorescence with an operating microscope. Therefore, we stored ex vivo ultrasonic tissue fragments in a low-volume incubation chamber (Scientific Systems Design) and bathed the tissue fragments in 1 mM 5-ALA (Sigma: 5451-09-2) for 30 min prior to experiments, which was continued during experiments.^21^

The ultrasonic samples were imaged on an interface recording chamber^22^ (Scientific Systems Design), which was mounted on an Olympus BX51WI epifluorescence microscope. A custom filter set (excitation 402/15 nm, emission 654/75 nm; Chroma Technology Corp.) was fitted to the filter cube turret of the BX51WI microscope to detect PpIX fluorescence. The laboratory fluorescence images and brightfield images were acquired on a Spot RT sCMOS cooled 5MP camera (RT39M5, Spot Imaging) controlled by Spot Advanced imaging software (Spot Imaging).

The fluorescence signal was quantified in FIJI (https://imagej.net/Fiji).^23^ The mean pixel intensity of the background (no tissue) was subtracted from pixel intensity in the tissue fragment region of interest. The pixel intensities in the region of interest were summed and divided by the area of the region of interest to give a total fluorescence signal × 10^7^ per mm^2^, which is expressed in relative fluorescence units (RFU).

### Neuropathological Processing

Ex vivo ultrasonic samples from the edge and core of high-grade gliomas were imaged in the laboratory in 5 participants. Both the core and edge samples were then processed histologically for tumor markers. The tissue fragments were fixed in 10% formalin and embedded in paraffin blocks; 4–5 µm sections were cut for immunohistochemistry. Immunohistochemistry, diagnostic genetic testing, and neuropathological assessment of brain tumors were performed by the Department of Clinical Neuropathology, King’s College Hospital, which is accredited by the United Kingdom Accreditation System and works in accordance with ISO standards 15189 for medical laboratories.

Neuropathology slides were imaged on an Olympus Slide scanner VS120. The images were analyzed with FIJI software (https://imagej.net/Fiji). A circularity and size filters were applied to the identified objects in the image. The filter parameters were adjusted to select rounded tumor cells and to discard nestin-positive endothelial cells, which were more elongated. The methylation status of the *MGMT* gene promoter at 4 CpG sites was determined by pyrosequencing using the *therascreen* MGMT pyro kit (Qiagen).

### Statistical Analysis

Graph Pad Prism 8 was used for statistical analysis and graphing. Normally distributed data were described by their mean ± standard error. The linear regression function was used to fit lines to data. Statistical tests were two-tailed and had a threshold for type 1 statistical error of *α* < 0.05. Means were compared with *t*-tests or a repeated-measures ANOVA (*MGMT* gene promoter methylation) if the data fulfilled the assumptions for parametric tests. A Mann–Whitney *U*-test was used when parametric tests were not appropriate. The power relationship between cell proliferation, measured by the density of Ki-67-positive nuclei, and tumor infiltration was assumed to be:

$$\begin{array}{l} Ki\text{-}67\text{-}positivenuclei=k{\left(tumorinfiltration\right)}^{n} \end{array}$$

where k is a constant and *n* is the power value. The data were plotted in the log–log format (Figure 4F) so that the slope of the line estimates the power, *n*, and the intercept with the *x*-axis when log Ki-67-positive nuclei equals zero (dashed line, Figure 4F) gives a measure of the number of tumor cells that are present when one cell is proliferating.

## Results

The presence of glioma cells in the screened ultrasonic tissue fragments was confirmed with histochemical stains and immunocytochemical stains against brain tumor markers (Figure 1).

### Ultrasonic Tissue Fragments Exhibit Histopathological Features of Gliomas

**Fig. 2:**
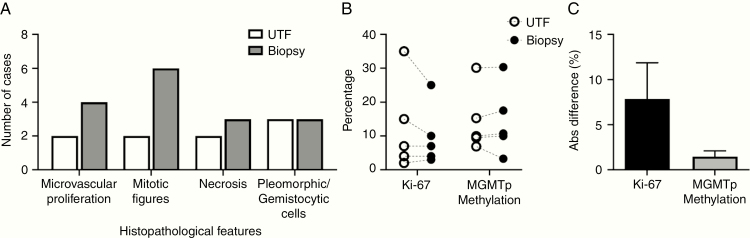
Surgical biopsy compared with ex vivo tumor-core ultrasonic tissue fragments (UTFs) from the same gliomas. (A) Glioma histopathology features found in surgical biopsies compared with tumor-core UTFs. (B) Comparison of the results from the surgical biopsy and tumor-core UTFs for the percentage of cells expressing nuclear Ki-67 staining and for percentage methylation of MGMT gene promoter (MGMTp) levels analyzed from individual gliomas. (C) Mean absolute percentage difference in Ki-67 and MGMTp methylation between the surgical biopsy and tumor-core UTFs for each participant. Error bars denote standard error of the mean.

The World Health Organization (WHO) grading of brain tumors is based on the histopathological and molecular features of the tumors.^1^ We first investigated the histopathological features that were exhibited by ultrasonic tissue fragments from WHO grade III–IV gliomas. A consultant neuropathologist (I.B.) examined the ultrasonic tissue fragments for histopathological features of cell division (mitotic figures), vascular proliferation, necrosis, and presence of pleomorphic cells or gemistocytes and compared the findings with the results from the tumor biopsy. The probability of the ultrasonic samples exhibiting a histopathological feature found in the tumor biopsy was 0.33–1.0 (*n* = 7 gliomas; Figure 2A, Table 1).

**Table 1.** Neuropathological Comparison of Ultrasonic Tissue Fragments and Surgical Biopsy

	Neuropathology—WHO Features	
	Ultrasonic tissue fragments	Biopsy
A	• No microvascular proliferation	• Perineuronal satelitosis
	• No mitotic figures	• No endothelial hyperplasia
	• No necrosis	• No necrosis
B	• Mitotic activity	• High mitotic activity
	• Microvascular proliferation	• Microvascular proliferation
	• No necrosis	• Apoptotic bodies
		• Pseudo-palisading necrosis
C	• Mitotic activity	• Mitotic activity
	• Microvascular proliferation	• Microvascular proliferation
	• Necrosis	• Necrosis
D	• Pleomorphic spindles	• Pleomorphic spindles
	• No mitotic activity	• Mitotic activity
	• No microvascular proliferation	• Microvascular proliferation
E	• Gemistocytic cells	• Gemistocytic cells
	• No mitotic activity	• Occasional mitotic activity
	• No microvascular proliferation • No necrosis	• No microvascular proliferation • No necrosis
F	• Necrosis	• Necrosis
	• Pleomorphic cells	• Pleomorphic cells
	• No microvascular proliferation	• Microvascular proliferation
	• No mitotic activity	• No mitotic activity
G	• No necrosis	• No necrosis
	• Pleomorphic cells	• Pleomorphic cells
	• No microvascular proliferation	• Perinuclear halo formation
	• No mitotic activity	• Mitotic activity

We found that a tumor diagnosis could be made from the histopathological features exhibited by the ultrasonic samples in 6/7 cases. In one case, there were too few glioma cells in the ultrasonic samples to reach a diagnosis. We concluded that large ultrasonic tissue fragments could give histopathological information about the tumor if the tissue fragments were taken from the body of the tumor and avoided large areas of necrosis as reported previously.^9,10,24^

### Molecular Markers in Ultrasonic Tissue Fragments

Molecular markers have become increasingly important to the diagnosis and management of gliomas.^1^ We asked whether ultrasonic tissue fragments provided quantifiable information about molecular markers. We first investigated markers of cellular proliferation in the ultrasonic samples by studying the expression of the Ki-67 protein in the nucleus (Figure 1E). We found that the percentage of cells expressing Ki-67 in the ultrasonic samples from the tumor core was within 7.9 ± 4.0% (*n* = 6 gliomas) of the Ki-67 expression in the tumor biopsy (Figure 2B and C).

We next studied the methylation status of the promoter region of the *MGMT* gene. The MGMT protein is important therapeutically because it removes the alkyl groups on guanine bases that are added by the chemotherapy drug, temozolomide. Methylation of the CpG dinucleotides in the promoter of the *MGMT* gene reduces the expression of the MGMT protein, which enhances the cytotoxicity of temozolomide. The average percentage methylation of the *MGMT* gene promoter in the ultrasonic tissue fragments taken from the core of the tumor was comparable to the values for the diagnostic biopsy (mean percentage difference 1.5 ± 0.6%, *n* = 5 gliomas; Figure 2B and C). We concluded that ultrasonic samples can provide quantifiable information about molecular markers relevant to the diagnosis and management of gliomas.

### Fluorescence Imaging to Screen Ex Vivo Ultrasonic Tissue Fragments for Glioma Infiltration

PpIX fluorescence was seen intraoperatively with the operating microscope in 4/7 participants (0/1 WHO grade II, 1/3 WHO grade III, 3/3 WHO grade IV; Table 2). PpIX fluorescence was seen in the ultrasonic samples from 6/7 participants. In one participant with anaplastic oligodendroglioma (case G, Table 2), no fluorescence was seen intraoperatively or during the screening of ultrasonic samples. In this case, very few glioma cells were present in the ultrasonic samples. We concluded that screening of ultrasonic tissue fragments with PpIX can detect infiltration by high-grade glioma cells at lower levels than seen during surgery.

**Table 2.** Clinical Information

	Age (Years)	Sex	Classification	Location	Intraoperative PpIX Fluorescence
A	27	F	Oligodendroglioma, IDH1-mutant, grade II	Left frontal/insula	Negative
B	62	M	Glioblastoma, IDH wildtype, grade IV	Right temporal	Positive
C	72	M	Glioblastoma, IDH wildtype, grade IV	Left parietal	Positive
D	29	F	Astrocytoma, IDH1-mutant, grade III	Left parietal	Positive
E	71	F	Astrocytoma, IDH1-mutant, grade III	Right temporal	Negative
F	64	M	Glioblastoma, IDH wildtype, grade IV	Right temporal	Positive
G	71	M	Oligodendroglioma, IDH1-mutant, grade III	Right frontal	Negative

We next explored whether ultrasonic samples could contribute to the evaluation of heterogeneity within a glioma. Histopathological screening for tumor infiltration in each fragment before molecular analysis would not be sustainable due to the large number of fragments collected during the surgery. Therefore, we developed a method to screen ex vivo ultrasonic tissue fragments for glioma infiltration using fluorescence imaging (see the Materials and Methods section).

**Fig. 3:**
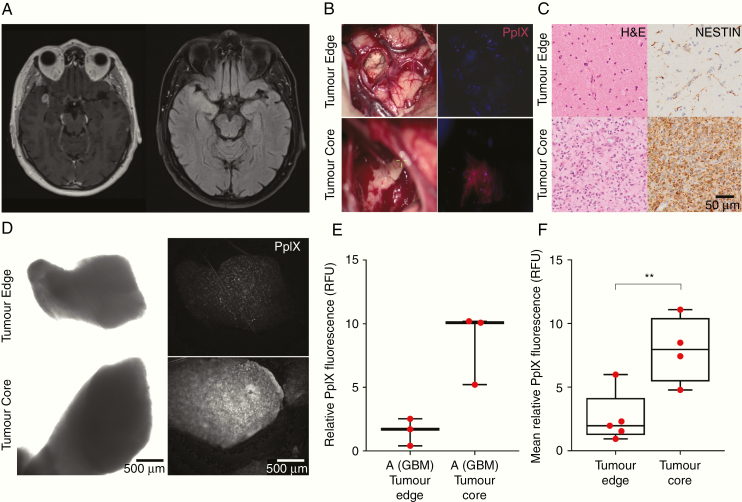
Screening for glioma infiltration in ex vivo ultrasonic tissue fragments (UTFs) with PpIX fluorescence. (A–E) Data from one participant. (A) Contrast-enhanced T1-weighted and FLAIR MRI scans showing a contrast-enhancing lesion in the anterior right temporal lobe. (B) Intraoperative brain surface images (right, brightfield; left, PpIX fluorescence) of tumor edge (top panels) and tumor core (bottom panels). (C) H&E (left panels) and Nestin (right panels) staining of sections through UTFs collected from the tumor edge (upper panels) and the tumor core (lower panels) showing marked glioma infiltration in the tumor core UTFs and sparse glioma infiltration of the tumor-edge UTFs. (D) Brightfield images (left panels) and PpIX fluorescence images (right panels) of ex vivo UTFs from tumor edge (top panels) and tumor core (bottom panels). (E) Relative PpIX fluorescence from the tumor edge (*n* = 3 UTFs, mean 1.6 RFU) and tumor core (*n* = 3 UTFs, mean 8.5 RFU; *P* = .062, paired *t*-test) of one glioma. (F) Mean relative PpIX fluorescence from UTFs collected from the tumor edge (*n* = 5 gliomas) and tumor core (*n* = 4 gliomas) (*P* = .01, *t*-test).

We took advantage of the 5-ALA-induced fluorescence that is used by neurosurgeons to image WHO grade III–IV gliomas during surgery.^15^ 5-ALA is metabolized in cells to Protoporphyrin IX (PpIX), which accumulates in glioma cells and causes them to be fluorescent (Figure 3A and B). For tumors exhibiting PpIX fluorescence during surgery, the core was defined as a brain region where a fluorescence signal was visible with the operating microscope whereas the tumor edge exhibited no fluorescence (Figure 3B and C; see the Materials and Methods section).

PpIX fluorescent cells could be seen in ex vivo ultrasonic tissue fragments taken from the tumor edge. The relative PpIX fluorescence emitted by ex vivo ultrasonic tissue fragments from the tumor core was higher than from the tumor edge (mean relative PpIX fluorescence: core = 8.0 ± 1.3 RFU, *n* = 4; edge = 2.5 ± 0.9 RFU, *n* = 5; *P* = .010, *t*-test; Figure 3D–F).

**Fig. 4:**
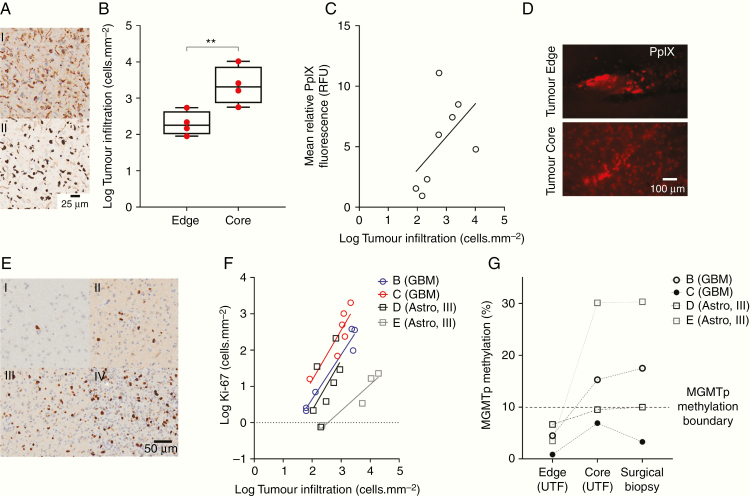
Heterogeneity of cell proliferation and MGMTp methylation of gliomas. (A) Immunocytochemically labeled glioma cells identified using a circularity filter. Top panel, image (×20) of nestin-stained section. Lower panel, cell bodies of nestin-positive cells labeled in black after analysis. (B) Density of tumor infiltration in UTFs from the edge and core of gliomas that were either nestin-positive or IDH1 (R132H) mutation positive (*n* = 4 gliomas; *P* = .04, paired *t*-test). (C) Relationship between mean PpIX relative fluorescence and mean tumor infiltration of UTFs collected from tumor core and edge (*n* = 4 gliomas, *r*^2^ = 0.28, *P* = .18). (D) Clustering of cells exhibiting PpIX fluorescence in ex vivo UTFs from the tumor edge and core. (E) Variation in Ki-67-positive cells in different UTFs from one participant. (F) Plot showing power relationship between density of Ki-67-positive cells and tumor infiltration. Data from UTFs collected from 4 gliomas (slope for glioma: A = 1.2, *r*^2^ = 0.96, *P* < .001; C = 1.4, *r*^2^ = 0.79, *P* = .018; F = 1.4, *r*^2^ = 0.34, *P* = .17; G = 0.72, *r*^2^ = 0.90, *P* = .06). (G) Variation in methylation status of the MGMT gene promoter from the 4 gliomas in F. UTFs from tumor edge and core are compared with the surgical biopsy.

We calculated a second, separate immunocytochemical measure of tumor infiltration by quantifying high-grade glioma cells that were positive for either nestin or for IDH1 (R132H) mutant protein at the core and tumor edge (Figure 4A and B). Ultrasonic tissue fragments from the core of the tumor had a higher density of glioma cells than the edge (core = 2512 cells/mm^2^, edge = 200 cells/mm^2^, *n* = 4 gliomas, *P* = .015 paired *t*-test; Figure 4B, data logged for presentation).

We then compared the relationship between relative PpIX fluorescence imaging and tumor infiltration measured immunocytochemically. There was not a tight relationship between the relative PpIX fluorescence signal and the extent of tumor infiltration (*R*^2^ = 0.28, *P* = .18, *n* = 8; Figure 4C). We explored this further by studying the spatial distribution of PpIX fluorescent cells. The tumor core was densely labeled with PpIX fluorescent cells (Figure 4D). At the tumor edge, the PpIX fluorescent cells were not evenly distributed, but were frequently found clustered together (Figure 4D).

We concluded that ex vivo ultrasonic tissue fragments could be screened for tumor infiltration with fluorescence imaging. The screening can be done rapidly because the tissue fragments do not need resectioning. The tendency for PpIX fluorescent cells to form small cell clusters made it easier to detect low levels of tumor infiltration at the tumor edge.

### Ex Vivo Ultrasonic Samples to Assess Tumor Heterogeneity

The ex vivo ultrasonic tissue fragments exhibiting PpIX fluorescence were used to study heterogeneity within high-grade gliomas.

We first asked whether ex vivo ultrasonic tissue fragments gave information on variability in cell proliferation in different parts of the high-grade gliomas. We stained ex vivo ultrasonic samples from different parts of a tumor for Ki-67 to measure cell proliferation (Figure 4E) and immunocytochemical tumor markers to quantify tumor infiltration. We found that cell proliferation, measured by the density of Ki-67-positive nuclei, had a power relationship with tumor infiltration for each glioma (Figure 4F; see the Materials and Methods section). The power values for high-grade gliomas (WHO grade III–IV) ranged from 0.7 to 1.4 (Figure 4F). The lines from the more aggressive glioblastomas were to the left of the anaplastic astrocytomas (Figure 4F).

Next, we focused on the methylation status of the MGMT gene promoter. The mean value for the methylation status of ex vivo ultrasonic tissue fragments from the core was similar to the values for the surgical biopsy (surgical biopsy = 15.3%, ex vivo ultrasonic samples, core = 15.5%, edge = 3.9%; repeated-measures ANOVA, *P* = .069) (Figure 4G). However, we found differences of up to 3.6%, which may be important in borderline cases (Figure 4G).

We concluded that ex vivo ultrasonic tissue fragments can capture variability in molecular markers, such as MGMT gene promoter methylation in the tumor core. Hence, ultrasonic tissue fragments can add to the surgical biopsy by giving information about tumor heterogeneity.

## Discussion

We investigated whether ex vivo ultrasonic samples of brain tissue can facilitate the diagnosis and treatment of gliomas. We show that large ex vivo ultrasonic tissue fragments can be screened rapidly with fluorescence imaging to detect tissue fragments infiltrated with a tumor. These tumor-infiltrated tissue fragments give detailed information about the molecular features of the tumor. This molecular information can be obtained either with immunocytochemical stains or through genetic sequencing. Importantly, ultrasonic tissue fragments give quantifiable data. Therefore, they can be used to characterize heterogeneity within a glioma. Consequently, ultrasonic samples can play a valuable role as an adjunct to brain tumor biopsy in both diagnosis and planning treatment.

Molecular profiling of ultrasonic tissue fragments would be easy to implement if there was a simple way to screen the tissue fragments for tumor infiltration. Fluorescence screening is the option we pursued. Ideally, the fluorescent label would only be expressed in glioma cells. One strategy is to use fluorophores that are actively concentrated within glioma cells. Therefore, we stored ultrasonic tissue fragments under ex vivo conditions to keep the brain tissue alive.^17,18^ We found that increased levels of PpIX in glioma cells can be used to detect tumor infiltration in ex vivo ultrasonic samples.

Tumor recurrence is a major problem in the management of brain tumors. A key issue is intratumor heterogeneity.^2,3,5^ The heterogeneity can be captured by collecting ultrasonic samples from different parts of the tumor.^25^ We found that individual gliomas manifested a power relationship between cellular proliferation, measured by Ki-67 expression, and tumor infiltration (Figure 4F). Higher-grade tumors showed greater cellular proliferation indices. Points above the line for a tumor indicate foci of higher than expected proliferation within the tumor and may serve as an early indicator of more malignant tumor behavior. The brain location of the tissue fragments can be recorded with the surgical neuronavigation system. Therefore, the collection site of tissue fragments with unexpectedly high proliferation indices can be identified. This information may be useful when planning treatment, such as radiotherapy.

The methylation status of the *MGMT* gene promoter is used to predict the response of gliomas to alkylating chemotherapy agents.^26^ Heterogeneity in the methylation status of tumor cells within individual gliomas has been described.^27,28^ The extent of heterogeneity in *MGMT* gene promoter methylation within gliomas requires further evaluation. It may be affected by several factors including the number of glioma cell clones in the tumor, tumor microenvironment, and developmental status of individual glioma cells. We found that screened ultrasonic tissue fragments capture the intratumoral heterogeneity. Notably, the *MGMT* gene promoter methylation in 1 of 4 participants was higher than reported from the surgical biopsy (Figure 4G). Hence, information from the ultrasonic tissue fragments gives a broader overview of methylation status within the tumor and may affect treatment decisions in cases of borderline methylation status.

Research studies have used the fluid from ultrasonic aspirators as a source of glioma cells for culturing and xenotransplantation into mice.^25,29,30^ Commonly, larger tissue fragments have been dissociated prior to culturing.^25^ Contamination of these cultures with healthy cells has been a concern in therapy studies.^30^ Fluorescence screening reduces this problem. Going forward, screened ex vivo fragments could be used directly in research involving 3D glioma models rather than being dissociated first.

The diagnosis and management of brain tumors are increasingly determined by the molecular profile of the tumor. This trend is accelerating. Tissue fragments from ultrasonic aspirators provide valuable molecular information that aids diagnosis. Treatment of brain tumors will increasingly be guided by actionable mutations in the tumor. Live ultrasonic tissue fragments are an underused source of tumor tissue that have great potential to improve how patients are diagnosed and treated.

## Funding

This work was supported by the Medical Research Council studentship [MR/N013700/1 to A.J.K.]; Psychiatry Research Trust and Inman Charity.

## 

**Conflict of interest statement.** A.J.K. is the CEO of Vivisco Limited. G.T.F. and A.K. are shareholders and sit on the Advisory Board. Vivisco did not contribute materials or funds to this project.

## 

**Authorship statement:** A.J.K., J.P.L., and G.T.F. designed the study. A.J.K., J.P.L., F.V., R.B., and K.A. collected samples and intraoperative images. A.J.K. did the experimental work. A.J.K. and G.T.F. analyzed the data. I.B. (consultant neuropathologist) made the neuropathological diagnoses. A.J.K., J.P.L., and G.T.F. wrote the manuscript with input from F.V., R.B., I.B., and K.A.
